# Protective capacity of carotenoid trans-astaxanthin in rotenone-induced toxicity in *Drosophila melanogaster*

**DOI:** 10.1038/s41598-022-08409-4

**Published:** 2022-03-17

**Authors:** Temitope C. Akinade, Oreoluwa O. Babatunde, Adeola O. Adedara, Olugbenga E. Adeyemi, Titilayomi A. Otenaike, Onaara P. Ashaolu, Titilayo O. Johnson, Ana Terriente-Felix, Alexander J. Whitworth, Amos O. Abolaji

**Affiliations:** 1 Drosophila Laboratory, Department of Biochemistry, Molecular Drug Metabolism and Toxicology Unit, Faculty of Basic Medical Sciences, College of Medicine, University of Ibadan, Ibadan, Nigeria; 2 Department of Biochemistry, Faculty of Basic Medical Sciences, College of Health Sciences, University of Jos, Jos, Nigeria; 3 Department of Physiology, Faculty of Basic Medical Sciences, College of Medicine, University of Ibadan, Ibadan, Nigeria; 4 MRC Mitochondrial Biology Unit, University of Cambridge, Cambridge, UK

**Keywords:** Biochemistry, Computational biology and bioinformatics, Neuroscience

## Abstract

Trans-astaxanthin (TA), a keto-carotenoid found in aquatic invertebrates, possesses anti-oxidative and anti-inflammatory activities. Rotenone is used to induce oxidative stress-mediated Parkinson’s disease (PD) in animals. We probed if TA would protect against rotenone-induced toxicity in *Drosophila melanogaster*. Trans-astaxanthin (0, 0.1, 0.5, 1.0, 2.5, 10, and 20 mg/10 g diet) and rotenone (0, 250 and 500 μM) were separately orally exposed to flies in the diet to evaluate longevity and survival rates, respectively. Consequently, we evaluated the ameliorative actions of TA (1.0 mg/10 g diet) on rotenone (500 μM)-induced toxicity in Drosophila after 7 days’ exposure. Additionally, we performed molecular docking of TA against selected pro-inflammatory protein targets. We observed that TA (0.5 and 1.0 mg/10 g diet) increased the lifespan of *D. melanogaster* by 36.36%. Moreover, TA (1.0 mg/10 g diet) ameliorated rotenone-mediated inhibition of Catalase, Glutathione-S-transferase and Acetylcholinesterase activities, and depletion of Total Thiols and Non-Protein Thiols contents. Trans-astaxanthin prevented behavioural dysfunction and accumulation of Hydrogen Peroxide, Malondialdehyde, Protein Carbonyls and Nitric Oxide in *D. melanogaster* (*p* < 0.05). Trans-astaxanthin showed higher docking scores against the pro-inflammatory protein targets evaluated than the standard inhibitors. Conclusively, the structural features of TA might have contributed to its protective actions against rotenone-induced toxicity.

## Introduction

Exposure to rotenone has been implicated in the pathophysiology of Parkinson’s disease (PD) in both animals and humans via mechanisms associated with oxidative stress and mitochondrial dysfunction. Indeed, exposure to rotenone extends beyond occupational setting as inadvertent non occupational exposures to rotenone via pesticides in the environment has been reported^1^. Rotenone is a member of rotenoids, that consists of a central dihydro-γ-pyrone ring which is proposed to be responsible for rotenone-mediated mitochondrial toxicity^2^. Rotenone is metabolized by CYP 3A4 or 2C19 to hydroxyl rotenone in the liver of rats and the mid-gut of insects^3^. Rotenone strongly inhibits mitochondrial complex I, blocks electron transport chain, increase electron leakage and produces energy deficit^4^. This results in excessive production of mitochondrial-ROS, which overcomes the capacity of the antioxidants system such as superoxide dismutase, catalase and glutathione peroxidase^5^. This then elicits oxidative damage to macromolecules and apoptosis of non-neuronal and neuronal cells^6,7^. The capacity of rotenone to penetrate Blood Brain Barrier (BBB) enables neuronal assault^7^. Additionally, rotenone inhibits microtubule assembly leading to mitotic arrest^7^. It also elicits the formation of cytosolic Lewy bodies and microglial activation in animals^8^.

Accumulation of Reactive Oxygen and Nitrogen Species (RONS) due to exposure to toxicants, can cause oxidative damage to biomolecular constituents of the cells and organelles^6,9^. Thus, search is ongoing for natural products with antioxidative and anti-inflammatory activities that can be used to prevent or reduce oxidative damage-induced diseases elicited by exposure to environmental contaminants.

Trans-astaxanthin (TA) is a natural red pigment (lipid-soluble keto-carotenoid member of xanthophylls) and precursor of vitamin A^10,11^. It can be commercially derived from lower plants (microalgae: *Haematococcus pluvialis*), aquatic animals (crustaceans and salmons etc.) or chemically synthesized^12^. It is also known as all-trans-astaxanthin, astaxanthin, E-astaxanthin, (S,S)-astaxanthin, astaxanthin or 3,3′-dihydroxy-β, β′-carotene-4,4′-dione^13^. It can exist as geometric- (cis or trans) or stereo- (Sinister or Rectus) isomers. The trans- and S-isomers are more stable than the Cis-isomer with higher bioactivity^14^. TA possesses much higher bioactivity than other carotenoids owing to its inherent polar ends [hydroxy (OH) and keto (C=O) on both terminal ends and nonpolar central moiety (polyene system or conjugated carbon–carbon double bonds (–C=C–C=C–)] in its chemical structure^12,15^. Therefore, the polyene system allows it to intercalate and also cross the BBB^16^. Both the polar and nonpolar moieties play significant roles in preventing the cells from oxidative damage by scavenging radicals, quenching singlet oxygen, inhibiting lipid peroxidation and regulating oxidative stress-mediated gene expression^17,18^. Furthermore, TA can form complexes with both lipoproteins and proteins to form carotenolipoproteins and carotenoproteins, respectively. It can diffuse through the BBB, thereby protecting the brain from acute injury and chronic neurodegeneration^19^. Interestingly, evidences of anti-inflammatory, immunomodulatory, antioxidant^20^ and neuroprotective activities^21^ of TA are available in the literature.

*Drosophila melanogaster* has been utilized as a model for testing the protective efficacy of natural compounds in the context of neurodegenerative diseases, including Parkinson’s disease model using rotenone-exposed or transgenic *Drosophila melanogaster*^22^. Drosophila has low or absent ethical limitations, short life cycle, high fecundity and low cost of maintenance^9,23,24^. In addition, it is being used to model human disease phenotypes, such as PD^22,25^. Chronic administration of rotenone to flies has been shown to cause selective degeneration of dopaminergic neurons, neuroinflammation and locomotor abnormality^26,27^. The use of TA to treat and attenuate the debilitating effects of rotenone-induced toxicity has not been reported. Therefore, in this study, we aimed to investigate the protective capacity of TA in rotenone-induced toxicity in flies as well as its molecular targets through molecular docking analyses.

## Results

### Effects of exposure to graded doses of TA on longevity, oxidative stress status and acetylcholinesterase activity in *D. melanogaster*

**Figure 1:**
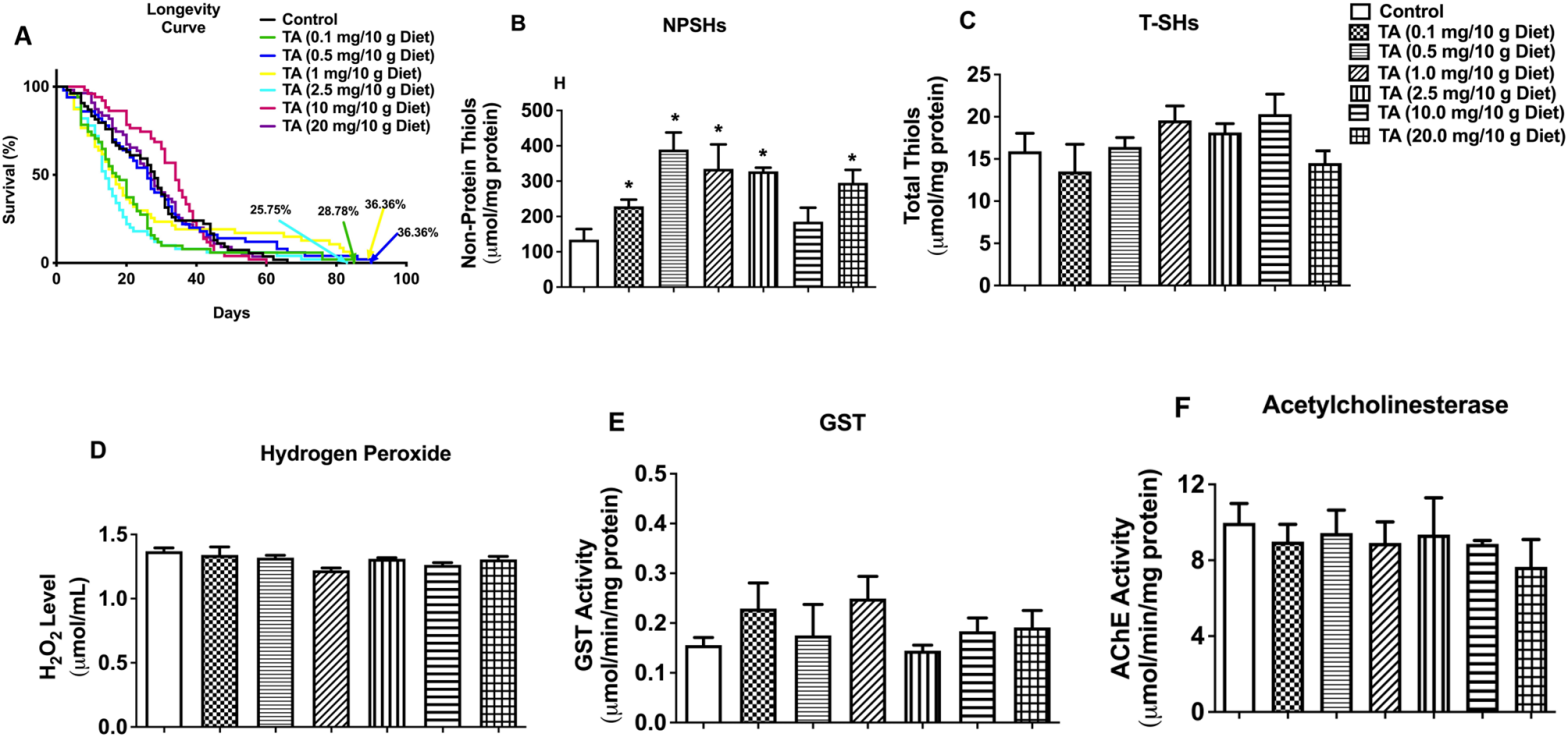
Effects of TA (0.1, 0.5, 1.0, 2.5, 10, and 20 mg/ 10 g diet) on longevity (**A**), levels of NPSHs (**B**), T-SHs (**C**) and H_2_O_2_ (**D**), and activities of the enzymes GST (**E**) and AChE (**F**) in *D. melanogaster*. Data in (**B**–**F**) are presented as mean ± SEM of 50 flies per vial (five replicates per group). *Significant difference compared with control group (p < 0.05). *TA* trans-astaxanthin, *NPSHs* non-protein thiols, *T-SHs* total thiols, *GST* glutathione-*S*-transferase, *AChE* acetylcholinesterase.

The TA concentrations of 0.5 and 1.0 mg/10 g diet both increased longevity by 36.36% compared to the control (Fig. 1A). Seven days of exposure of flies to TA indicated no adverse effects on NPSHs (Fig. 1B), T-SHs (Fig. 1C), H_2_O_2_ (Fig. 1D), GST (Fig. 1E) and AChE (Fig. 1F). In addition, TA significantly boost the level of NPSHs compared with the control (p < 0.05). Considering the fact that 0.5 and 1.0 mg/10 g diet concentrations of TA induced the highest effect on the lifespan of flies than the other concentrations, we selected 1.0 mg/10 g of diet concentration to investigate its protective role in rotenone-induced toxicity in *Drosophila melanogaster.*

### Rotenone exposure impairs survival rate and alters antioxidative status and inflammatory marker in *D. melanogaster*

**Figure 2:**
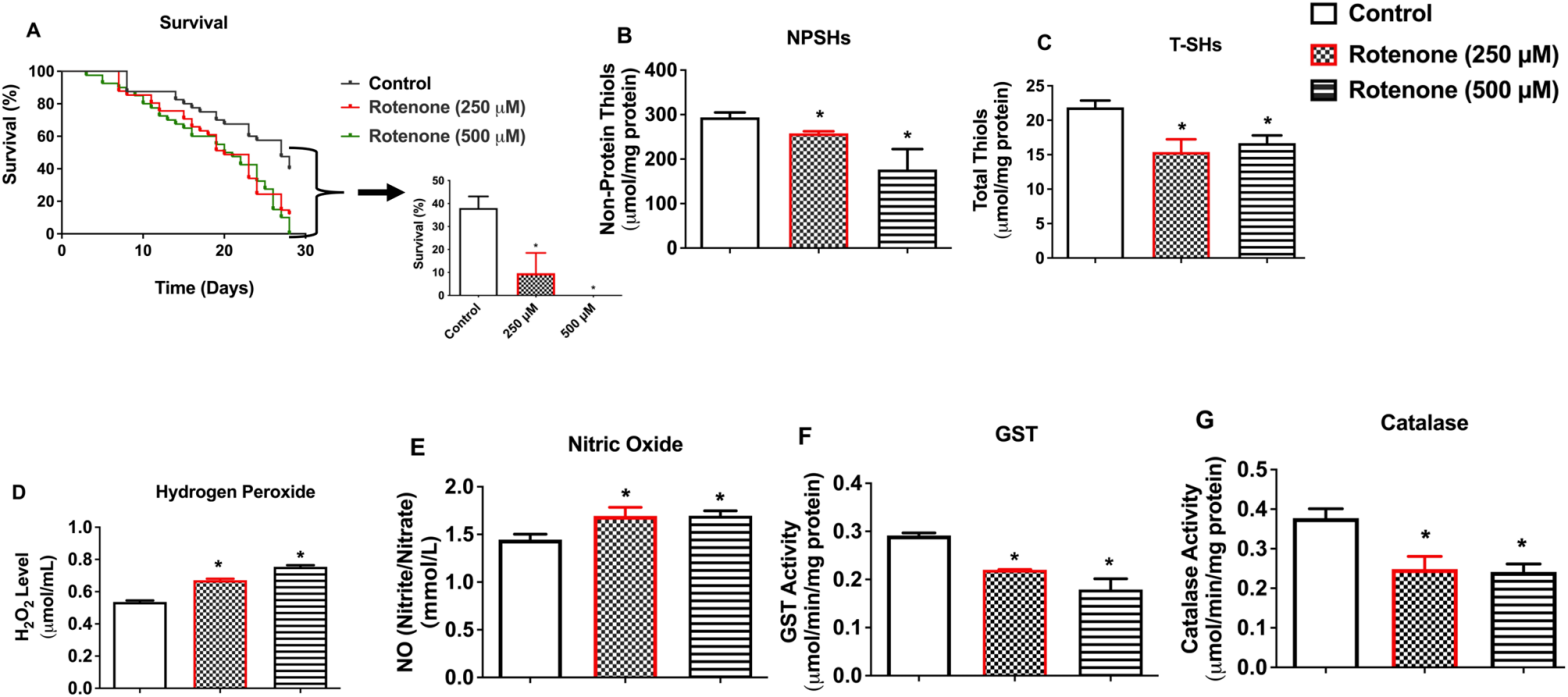
Effects of rotenone (250 and 500 μM) on 30-day survival rate (**A**), levels of NPSHs (**B**), T-SHs (**C**), H_2_O_2_ (**D**), NO (**E**), and activities of the enzymes GST (**F**) and Catalase (**G**) in *D. melanogaster*. Data in (**A**–**G**) are presented as Mean ± SEM of 50 flies per vial (five replicates per group). *Significant difference compared with control group (p < 0.05). *NPSHs* non-protein thiols, *T-SHs* total thiols, *H*_*2*_*O*_*2*_ hydrogen peroxide, *GST* glutathione-S-transferase, *AChE* acetylcholinesterase

Rotenone significantly reduced survival rate of *D. melanogaster* after 30 days of treatment compared with the control (Fig. 2A, p < 0.05). Additionally, rotenone significantly reduced NPSHs (Fig. 2B) and T-SHs (Fig. 2C) and elevated H_2_O_2_ (Fig. 2D) and NO (Fig. 2E) levels compared with the control. Apart from this, rotenone inhibited activities of GST (Fig. 2F) and Catalase (Fig. 2G) compared with the control flies (p < 0.05). Consequently, 500 μM dose of rotenone was selected in the ameliorative study with TA.

### TA attenuates rotenone-induced increased levels of oxidative stress and inflammatory markers, and reduced cell viability in *D. melanogaster*

**Figure 3:**
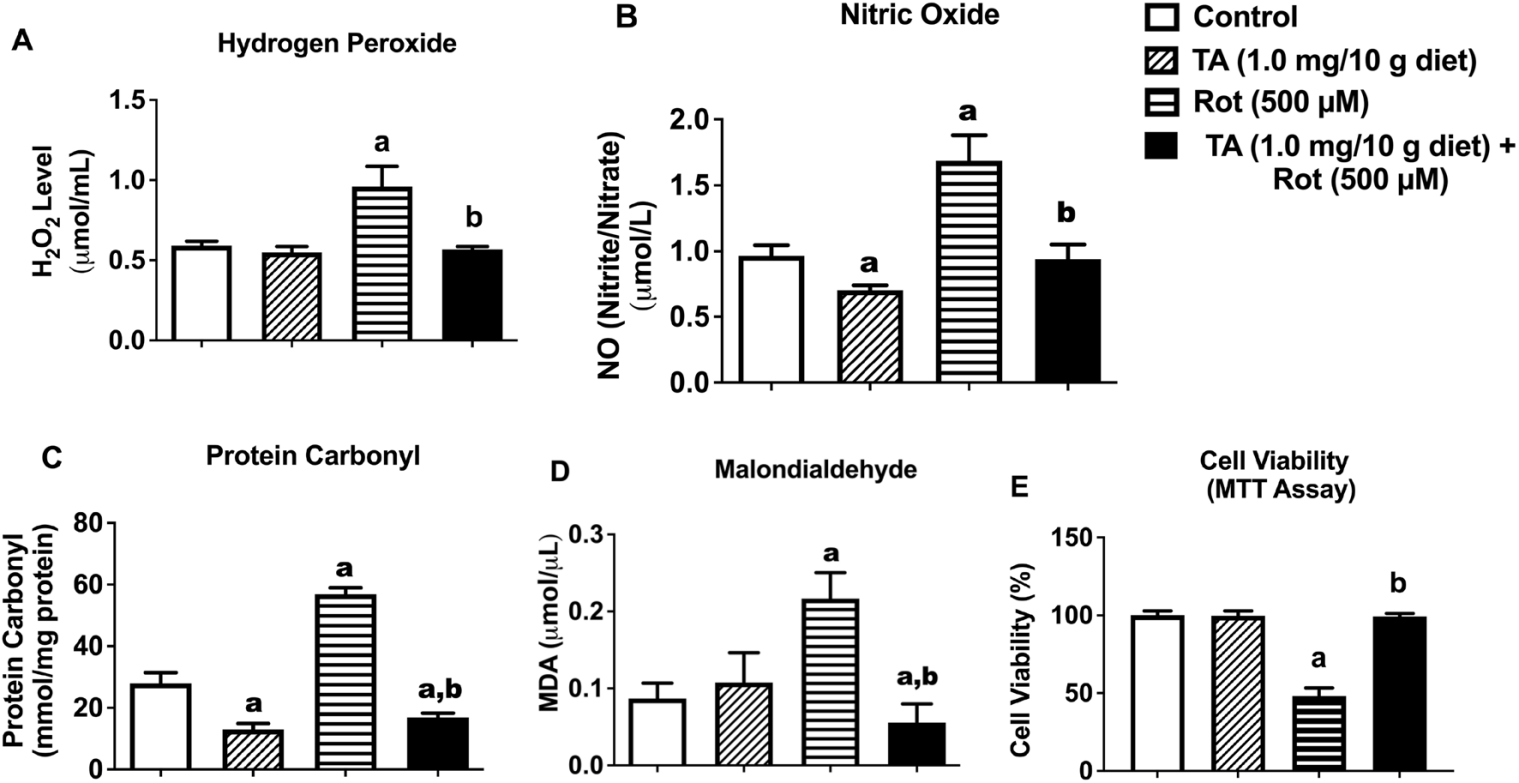
Effects of TA (1.0 mg/10 g diet) and/or rotenone (500 μM) on H_2_O_2_ (**A**), NO (**B**), Protein Carbonyl (**C**), Malondialdehyde (**D**), and Cell Viability (MTT assay) (**E**). Data in (**A**–**E**) are presented as Mean ± SEM of 50 flies per vial (five replicates per group). a: Significant difference compared with control group; b: Significant difference compared with rotenone group (p < 0.05). *TA* trans-astaxanthin, *H*_*2*_*O*_*2*_ hydrogen peroxide.

TA restored to normal, rotenone-induced elevation of H_2_O_2_ (Fig. 3A), nitric oxide (Fig. 3B, nitrite/nitrate), and blocked the increase of protein carbonyl (Fig. 3C), and malondialdehyde (Fig. 3D, p < 0.05). In addition, TA attenuated rotenone-induced reduction in mitochondrial metabolic rate (cell viability) assessed by MTT assay in *D. melanogaster* (Fig. 3E).

### TA Restores the antioxidant status impaired by rotenone in *D. melanogaster*

**Figure 4:**
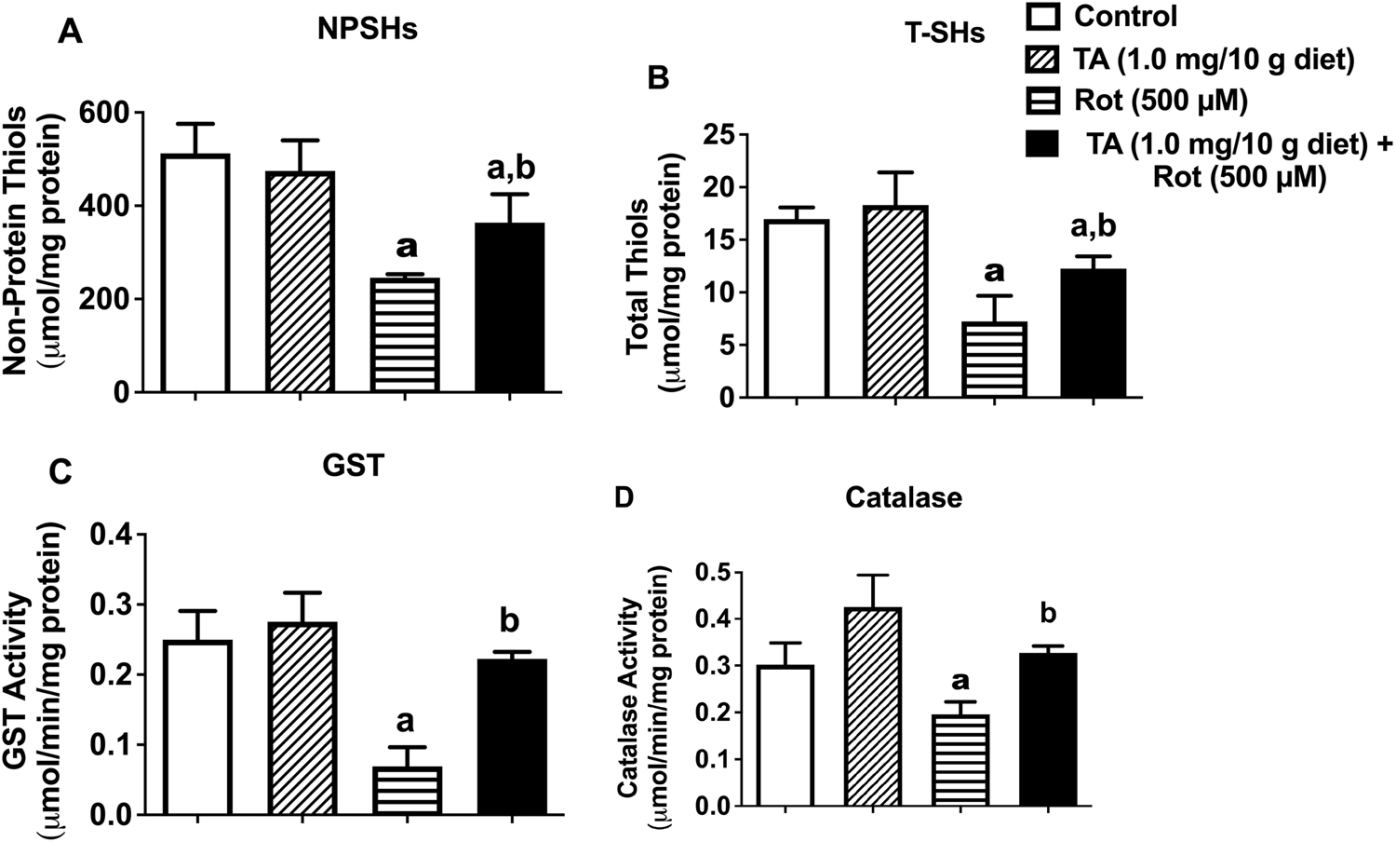
Effects of TA (1.0 mg/10 g diet) and/or rotenone (500 μM) on Levels of NPSHs (**A**), T-SHs (**B**), and activities of the enzymes GST (**C**) and Catalase (**D**). Data in (**A**–**D**) are presented as Mean ± SEM of 50 flies per vial (five replicates per group). a: Significant difference compared with control group; b: Significant difference compared with rotenone group (p < 0.05). *TA* trans-astaxanthin, *NPSHs* non-protein thiols, *T-SHs* total thiols, *GST* glutathione-S-transferase.

The effects of rotenone and TA on NPSHs, T-SHs, catalase and GST are shown in Fig. 4. Indeed, TA partially prevented rotenone-induced depletion of NPSHs (Fig. 4A) and T-SHs Fig. 4B) contents and ameliorated rotenone-induced inhibition of GST (Fig. 4C) and catalase (Fig. 4D) activities in *D. melanogaster.*

### TA improved behavioral dysfunction and acetylcholinesterase activity and did not alter reduced emergence rate induced by rotenone in *D. melanogaster*

**Figure 5:**
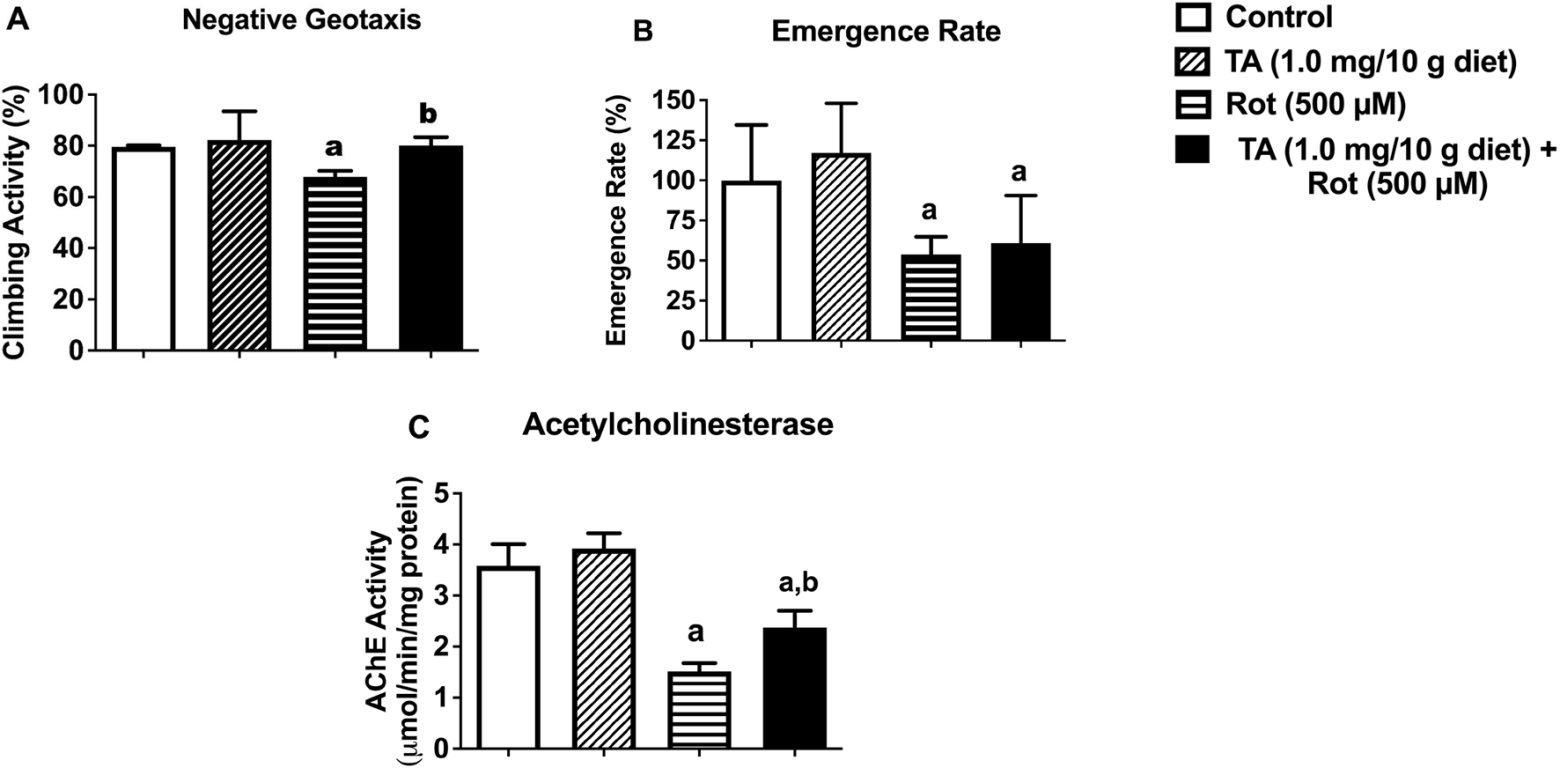
Effects of TA (1.0 mg/10 g diet) and/or rotenone (500 μM) on emergence rate (**A**), and acetylcholinesterase activity (**B**). Data in (**A**–**C**) are presented as Mean ± SEM of 50 flies per vial (five replicates per group). a: Significant difference compared with control group; b: Significant difference compared with rotenone group (p < 0.05). *TA* trans-astaxanthin.

The effects of rotenone and TA on negative geotaxis (locomotor activity), emergence rate and AChE are shown in Fig. 5. We found that although TA ameliorated rotenone-induced behavioural deficit (Fig. 5A, p < 0.05), it could not rescue rotenone-induced reduction of emergence rate of progeny of flies (Fig. 5B, p > 0.05). In addition, TA partially attenuated rotenone-induced inhibition of AChE activity (Fig. 5C).

### Molecular docking analysis

The docking scores of TA was higher than those of the standard inhibitors (Rofecoxib, Pralnacasan and Thalidomide) for the inflammatory protein targets (TNF-α, Caspase-1 and Cox-2) in both *D. melanogaster* and *H. sapiens*, except for *Drosophila* capase-1 (DmCasp-1) where the docking score of Pralnacasan (− 7.8 kcal/mol) was lower compared with that of TA which was − 7.2 kcal/mol (Table 1).

**Table 1:** Binding affinities of trans-astaxanthin (TA) and some standard inhibitors for the selected anti-inflammatory protein targets.

Standard nhibitors	PubChem CID	∆G energy (Kcal/mol)					
		DmTNF-α	HsTNF-α	DmCasp-1	HsCasp-1	DmCox-2	HsCox-2
TA	5281224	− 6.8	− 7.7	− 7.2	− 8.3	− 7.6	− 7.3
Pralnacasan	153270			− 7.8	− 8.1		
Rofecoxib	5426	− 6.0	− 6.5				
Thalidomide	5090					− 5.8	− 6.6

Dm: *Drosophila melanogaster*; hm: human, TNF-α: tumour necrosis factor-alpha (Eiger in *Drosophila*), Caspase-1 (Dcp-1 (*Drosophila* caspase-1) in *Drosophila*); and Cox-2 (COII (cytochrome oxidase subunit 2) in *D. melanogaster*).

**Figure 6:**
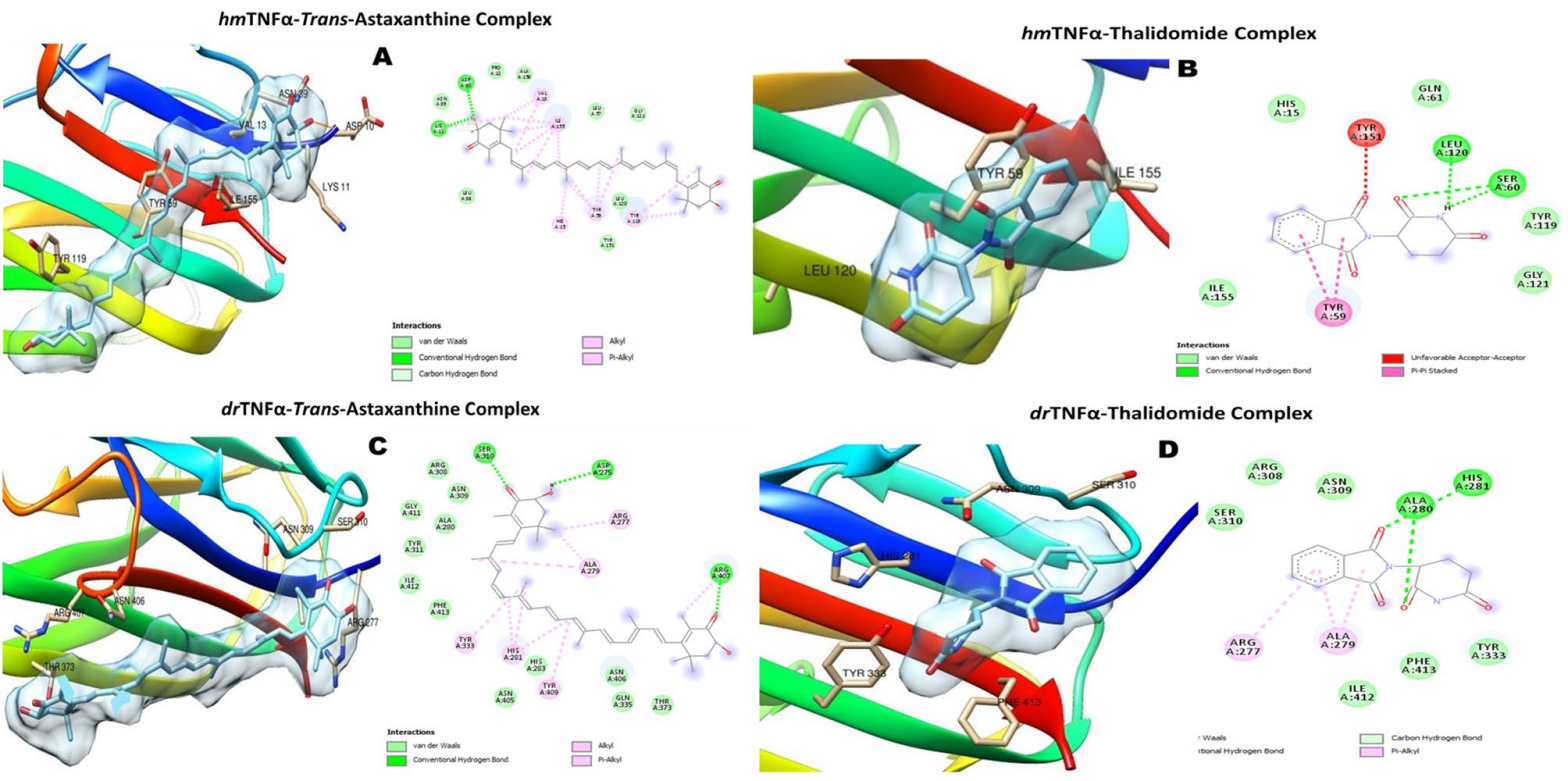
The 3D and 2D molecular complexations of Human TNFα (hmTNFα) with TA (**A**) and Thalidomide (**B**), and Drosophila TNFα (drTNFα) with TA (**C**) and Thalidomide (**D**) respectively.

Also, TA occupied the binding pockets of the respective standard inhibitors for all the protein targets, thus, sharing similar amino acids for interactions at the active sites (Fig. 6).

**Figure 7:**
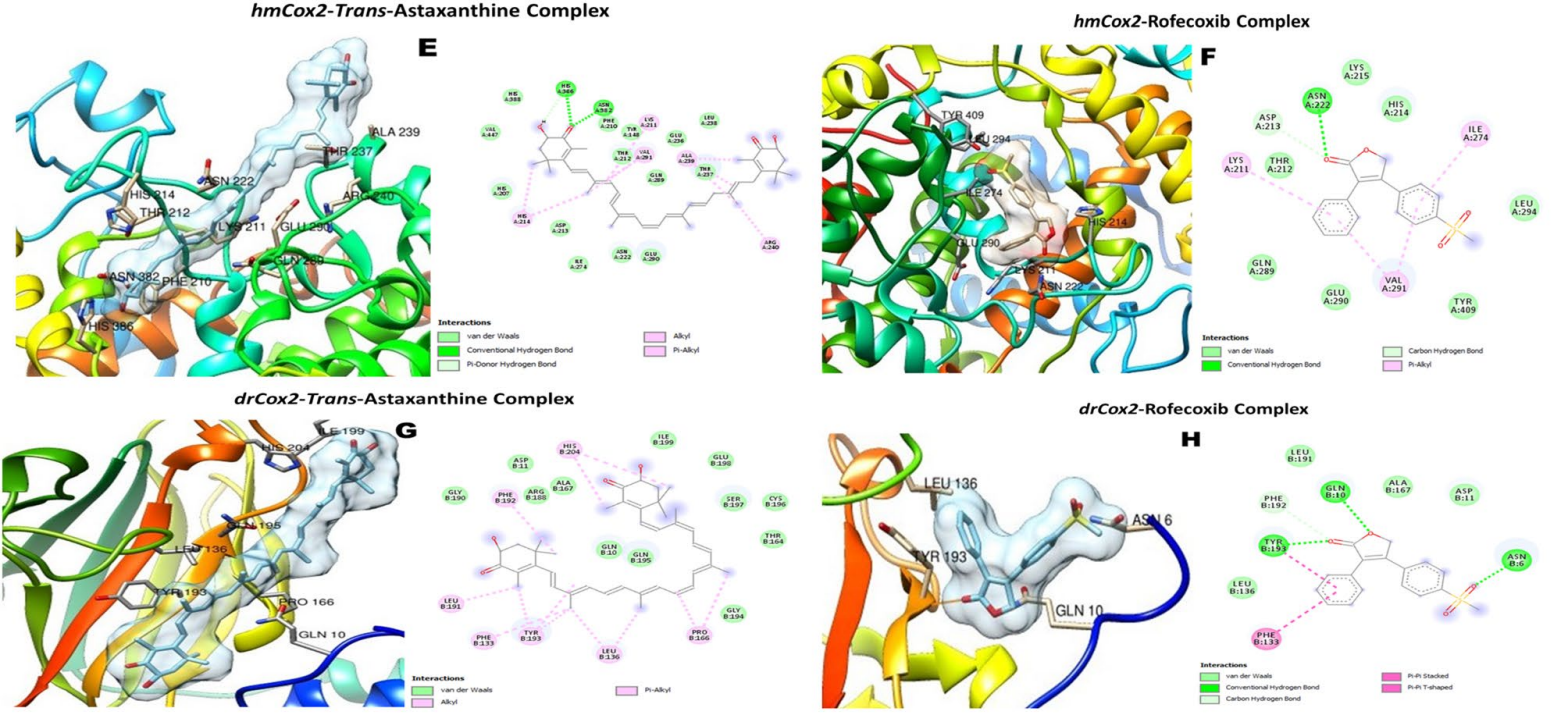
The 3D and 2D molecular complexations of Human Cox2 (hmCox2) with TA (**E**) and Rofecoxib (**F**); and *Drosophila* Cox-2 (drCox-2) with TA (**G**) and Rofecoxib (**H**) respectively.

**Figure 8:**
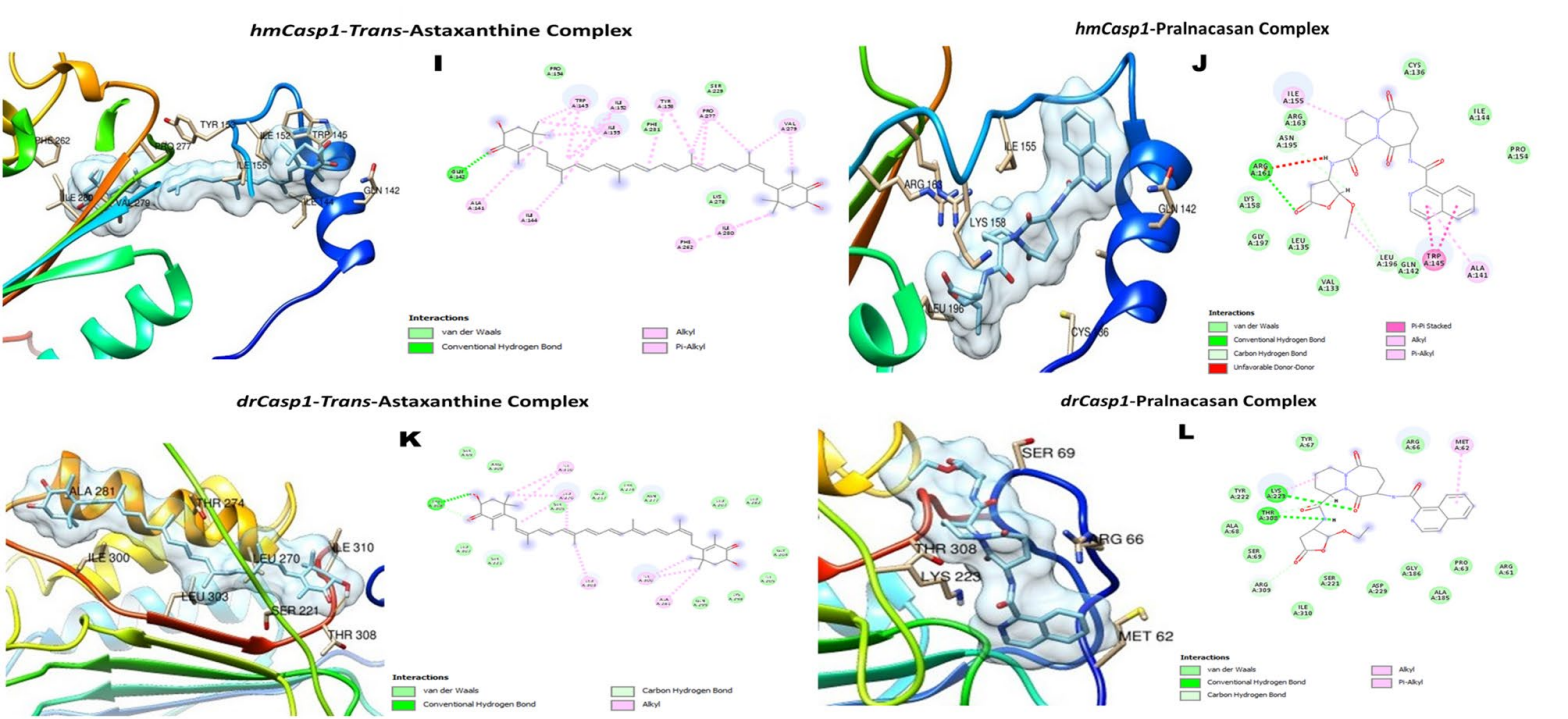
The 3D and 2D molecular complexations of Human Caspase-1 (hmCasp-1) with TA (**I**) and Pralnacasan (**J**); and *Drosophila* Caspase-1 (drCasp-1) with TA (**K**) and Pralnacasan (**L**) respectively.

TA and Thalidomide interacted with the triad amino acid residues (His 281, Ala 279 and Arg 277) of the active site of the DmTNF-α. Both compounds also interacted with common amino acid residues at the active pocket of HsTNF-α (Fig. 6A–D). Both TA and Rofecoxib bound to Val 291 and Lys 211 residues of the active pocket of HsCox-2, and to Tyr 193, Phe 192 and Phe 133 triad residues of the *Drosophila* homolog of the same enzyme (Fig. 7E–H). The interactions of the natural compound and the caspase-1 inhibitor (pralnacasan) at the binding pockets of the human protein target (HsCasp-1) occurred with Ile 155, Trp 145 and Ala 141 residues; and of the *Drosophila* homologue (DmCasp-1) with Thr 308 residue (Fig. 8I–L). Nonetheless, TA seemed to form strong hydrogen bonds, in addition to several other bond types (alkyl and van der Waal forces) (Figs. 6A–D,7E–H,8I,J).

## Discussion

Rotenone has been established to induce neurodegenerative diseases such as PD in animals including *Drosophila melanogaster*. Some of the hallmark features of PD include oxidative stress, inhibition of mitochondrial complex I, dopaminergic neuronal loss and locomotor abnormality^4,26,27^. However, the use of trans-astaxanthin (TA) to treat and attenuate the debilitating effects rotenone-induced toxicity in *D. melanogaster* is scarce in the literature. Here, we investigated the protective capacity of TA on rotenone-induced toxicity using *D. melanogaster* as a model. We evaluated different behavioural and oxidative stress markers as well as molecular mechanisms of actions of TA against inflammatory protein targets using in silico approach.

From the different concentrations of TA used in this study, 0.1, 0.5, 1.0 and 2.5 mg/10 g diet prolonged the lifespan of flies by 28.78, 36.36, 36.36 and 25.75%, respectively, compared with the control. This suggests that TA might possess anti-aging activity by extending the lifespans of the flies. Our finding is in support of the report of Huangfu et al*.*^28^ showing that TA has pro-longevity activity against *SOD1* null mutant flies by protecting against oxidative stress. It also corroborates the lifespan extension in *C. elegans* by preventing against oxidative stress through interaction with mitochondrial complex III electron leakage^29^.

Acetylcholine (ACh) and Dopamine (DA) are the major antagonistic neurotransmitters, released from the striatal cholinergic interneurons (SCI) and substantia nigra (SNi) neurons, respectively, onto the dorsal striatum of the midbrain^30^. Membrane-bound post-synaptic acetylcholinesterase (AChE) liberates choline and acetyl group from ACh into the synaptic cleft, thereby alleviating cholinergic toxicity that is responsible for the motor symptoms, such as bradykinesia, tremor and rigidity, observed in PD^31^. Several studies have indicated an altered AChE activity by rotenone in different animal models, including *D. melanogaster*^4,32^. In this study, we found that the inhibition of AChE activity by rotenone in *D. melanogaster*, was coupled with impaired locomotor activity. Interestingly, we found that TA improved the rotenone-induced inhibition of AChE activity and decline in locomotor performance of the flies.

The observed rotenone-induced accumulation of H_2_O_2_ and inhibition of catalase activity are indications of oxidative damage. Indeed, rotenone has been shown to inhibit mitochondrial complex I leading to electron leakage and superoxide radical production that in turn dismuted to H_2_O_2_ by superoxide dismutase. The elevated H_2_O_2_ can produce reactive and short-lived hydroxyl radical (OH·) through Fenton reaction, which in turn can induce oxidative damage to proteins, nucleic acids and lipid components^5^. These observations prompted us to corroborate the fact that, indeed, TA possesses antioxidative property against ROS^17,18^.

The observed inhibition of Glutathione-S-transferases (GST) in the rotenone-exposed flies might suggest GST’s dysfunctional detoxification capacity^9,33^. The observation that TA ameliorated rotenone-induced inhibition of GST further strengthens its antioxidative capacity. Consequently, we evaluated thiols’ status of the *D. melanogaster.* Thiols are carbon-bound sulfhydryls (SHs) chemical groups. They are classified into protein thiols and non-protein thiols (NPSHs), in which the latter is majorly composed of GSH, besides other thiols^34^. The thiols are involved in many physiological roles among which is tissue protection against oxidative damage^28^. Low level of thiols has been associated with many cellular dysfunctions, aging and neurodegenerative diseases, including PD^35,36^. Hence, decreased level of NPSHs has been reported in both PD patients and experimental models of PD^37^, including rotenone-induced PD model in flies^38^. In this study, TA restored the protective status of both total thiols and NPSHs, that were depleted in flies treated with rotenone.

We further evaluated other oxidative stress markers such as protein carbonyls (PCs) and malondialdehyde (MDA, a product of lipid peroxidation). In fact, different products of PCs are generated from oxidative assault to amino acid side chains or backbone cleavage in a protein by hydroxyl radicals^39^. PCs formed by the product of lipid peroxidation, such as MDA, has been used to accentuate the oxidative source of PCs^40^. Aging tissues and neurodegenerative diseases have been shown to be associated with increased levels of PCs and lipid peroxidation product^40^. Studies have reported elevated levels of PCs and MDA in *D. melanogaster* fed with rotenone^40^. Interestingly, we found that inclusion of TA in the diet reversed rotenone-induced elevation of PCs and MDA in flies.

Also, we sought to understand whether toxicity induced by rotenone reduced cell viability by carrying out MTT assay, which is a measure of cellular and mitochondrial metabolic rate. We found that rotenone indeed reduced cell viability in the flies. Strikingly, TA ameliorated rotenone-induced reduction of cell viability in *D. melanogaster*. Moreover, we carried out emergence rate of progenies of flies and found that TA did not attenuate rotenone-induced reduction in emergence rate.

Nitric oxide [NO, (nitrite/nitrates)] can react with superoxide anion radical to form a more poisonous oxidative species (ONOO·, peroxynitrite) that have been evidenced in many pathologies^41^. This pro-inflammatory marker is physiologically released during inflammatory burst by the immune cells. However, in diseased state, NO is uncontrollably released in an accumulative manner. This, consequently, can lead to the production of peroxynitrite and other pro-inflammatory markers that results in cellular apoptosis^42^. The remarkable amelioration of rotenone-mediated elevation of NO by TA confirms that it possesses anti-inflammatory activity.

Molecular docking analysis was performed to provide further insight into the molecular mechanism of the anti-inflammatory capacity of TA against rotenone-induced inflammation. Tumour necrosis factor-alpha (TNF-α), Cyclooxygenase-2 (Cox-2) and Caspase-1 (Casp-1) are the pro-inflammatory mediators accompanied with NO release, through NF-kB and inflammasome complex-mediated responses^43,44^. These proteins are members of the group of pro-inflammatory markers that might be stimulated or released by glial cells, as a result of the signalling interplays of their molecular origins, during chronic neuroinflammation-induced PD^45–47^. The Cox-2, TNF-α, and Caspase-1 have been implicated in the neuroinflammation-mediated PD^48^. The inhibition of these three protein targets are windows of opportunity for a new anti-PD drug development. In the light of this, TA was subjected to molecular docking against human TNF-α, Cox-2 and Casp-1 protein targets, as well as their *Drosophila* homologues. Interestingly, TA showed higher docking scores against both the human and *Drosophila* proteins than the standard inhibitors except for the Pralnacasan against DmCasp-1. The molecular interactions of TA with the anti-inflammatory protein targets could be the mechanisms employed by TA to regulate the inflammatory response triggered by exposure of flies to rotenone. The increased binding affinity of TA to target proteins can be attributed to different factors, such as the more negative change in free energy, the interaction of its different functional groups with the amino acid residues at the active pockets of the proteins, and the nature of the binding that occurred between them^49,50^. These molecular interactions depend on the chemical structure of TA which contains the keto- (C=O) and hydroxyl (OH–) groups on the two terminal β-ionone rings and the central conjugated carbon (–C=C–C=C–) group joining the two β-ionone rings. The C=O and OH– groups on the β-ionone ring formed different types of hydrogen bonds (conventional, carbon–hydrogen and pi-donor), being the strongest noncovalent bond, with the amino acids at the active sites of the human and *Drosophila* protein targets. Additionally, the polyene group, as well as free carbon–hydrogen (C–H) was also responsible for hydrophobic bond types such as alkyl and pi-alkyl. Likewise, van der Waal forces do exist. Thus, TA, using its hydroxyl group, was strongly hydrogen-bonded with Asp10 and Lys11 at the binding site of the human TNF-α, and also with Asp275, and Ser310 and Arg407, using hydroxyl and keto groups, respectively, at the binding site of the *Drosophila* homologue of TNF-α. Also, His386 and Asn382 at the binding pocket of human Cox-2 were tightly hydrogen bonded with both hydroxyl and carbonyl oxygen of the β-ionone ring of the TA, whereas it was the carbon–hydrogen of the ring and the conjugated bonds in the bioactive compound that formed several alkyl bonds with many amino acids at the active site of Cox-2 homologue of *D. melanogaster*. Nonetheless, many alkyl bonds and van der Waal forces of the carbon–hydrogen and polyene bonds in the TA with several amino acids in the binding site of the human Caspase-1 could also be responsible for the higher binding affinity.

**Figure 9:**
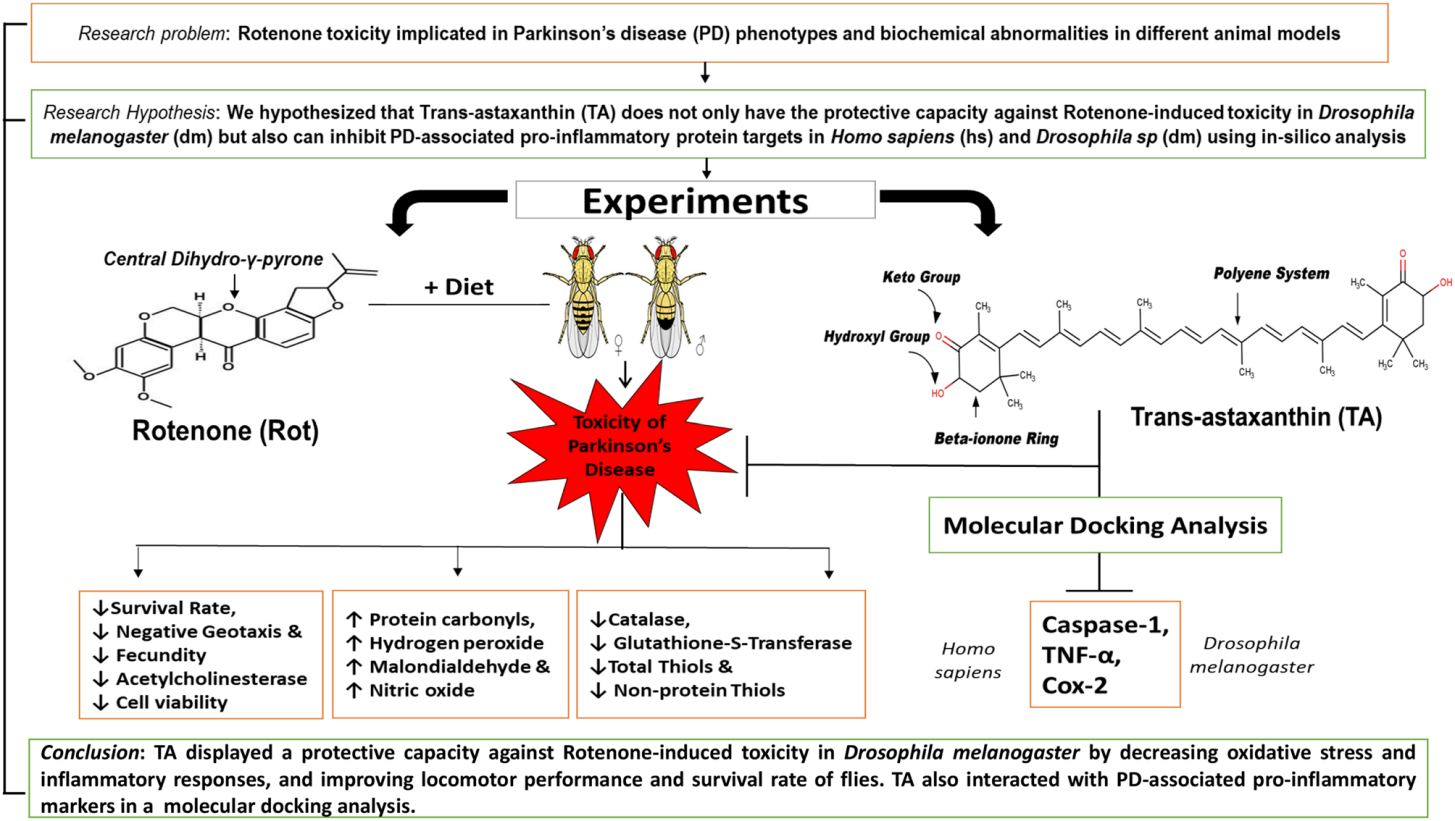
A flowchart showing an overview of experimental steps and outcome.

In conclusion, rotenone-induced toxicity is due to the perturbation of redox system, leading to oxidative stress, inflammation and behavioural abnormalities in flies. Interestingly, TA exhibited protection against rotenone-induced oxidative damage by enhancing endogenous antioxidant capacity, decreasing oxidative stress markers, and inflammation. Moreover, the interaction of the central polyene system as well as carbonyl and hydroxyl functional moieties of the two terminal β-ionone rings of TA, contribute to its antioxidative and anti-inflammatory properties (Fig. 9). Thus, TA can be considered as a promising therapeutic compound against oxidative stress and inflammation induced by rotenone and or related pesticides and insecticides.

## Materials and methods

### Chemicals

All chemicals used were commercial products of analytical grade. Trans-astaxanthin (TA) and rotenone were procured from AK Scientific, 30023 Ahern Ave, Union City, CA 94587, USA.

### *Drosophila melanogaster* stock and culture

*D. melanogaster* wild-type (Harwich strain) flies, that were obtained from National Species Stock Center (Bowling Green, OH, USA), were maintained and reared in *Drosophila* Laboratory, Biochemistry Department, University of Ibadan, Nigeria on cornmeal medium containing 1% w/v brewer's yeast, 2% w/v sucrose, 1% w/v powdered milk, 1% w/v agar, and 0.08% v/w nipagin at constant temperature and humidity (22–24 °C; 60–70% relative humidity) under 12 h dark/light cycle conditions.

### Treatment of *D. melanogaster* with TA and rotenone

In order to select suitable concentrations and duration of exposure of flies to TA and rotenone, we carried out longevity and 30-day survival assays, respectively. Based on the longevity and survival data obtained, 7 days was chosen as duration to evaluate individual effects of TA (0, 0.1, 0.5, 1.0, 2.5, 10, and 20 mg/10 g diet) and rotenone (0, 250 and 500 μM/10 kg diet) on some selected biochemical markers. Thereafter, the flies were anaesthetized on ice, weighed, homogenized in 0.1 M phosphate buffer (pH 7.4, ratio of 1 mg:10 μL) and centrifuged at 4000×*g* for 10 min at 4 °C in a thermo scientific Sorval Legend Micro 7R centrifuge. Supernatants were collected, stored at − 20 °C and used for the determination of the following biochemical parameters: Non-Protein Thiol (NPSHs), Total Thiol (T-SHs), Hydrogen peroxide (H_2_O_2_), Nitric Oxide (NO, nitrate and nitrate), catalase, Glutathione-S transferase (GST), and Acetylcholinesterase (AChE)^33^.

From the data obtained above, we selected 1.0 mg/10 g diet of TA to understand its protective capacity on rotenone (500 μM)-induced toxicity after 7 days of treatment as follows:

- Group 1 (Control, vehicle (2% Ethanol).
- Group 2: TA (1.0 mg/10 g diet (Ethanol vehicle).
- Group 3: Rotenone (500 μM).
- Group 4: TA (1.0 mg/10 g diet) + Rotenone (500 μM).

(50 flies/replicate, n = 5).

Thereafter, the flies were anaesthetized in ice, weighed, homogenized in 0.1 M potassium phosphate buffer (pH 7.4, 1 mg:10 μL buffer) and processed as described above for the determination of MTT assay and the following oxidative stress and antioxidant markers- H_2_O_2_, NPSHs, T-SHs, protein carbonyls, malondialdehyde (MDA), NO (nitrate and nitrite), catalase and GST as well as AChE anf emergence rate.

### Measurement of longevity and survival rate of *D. melanogaster* after exposure to TA and rotenone

Both sexes of *D. melanogaster* (Harwich strain) 1- to 3-day old, were divided into different groups (50 flies/replicate, n = 5), and treated separately with ethanol (2.0%, control) and TA (0.1, 0.5, 1.0, 2.5, 10, and 20 mg/10 g diet) and rotenone (0, 250 and 500 μM), respectively. The flies were changed at least once per week into diets containing similar concentrations of TA and rotenone. The flies were monitored and mortality was recorded daily, and used to plot the longevity curve for TA and 30 days survival rate for rotenone. The data were presented as a percentage of the control^9^.

### Measurement of locomotor activity

Locomotor activity of TA- and rotenone-exposed flies was determined by employing the method of Feany and Bender^26^. Briefly, ten flies from the control and treated groups were briefly immobilised using ice and separately placed in labelled glass column of 15 cm length and 1.5 cm in diameter. The flies were allowed to recover from anaesthesia and gently tapped to the bottom of the column. Thereafter, the number of flies that climbed up to the 6 cm mark and those below this mark were recorded. The data were then expressed as percentage of flies that crossed up to and beyond the 6 cm mark of the column.

### Biochemical parameters

#### Total protein determination

Total protein was determined according to the methods of Lowry et al*.*^51^ using Bovine Serum Albumin as standard. The values of the total proteins for samples were used to calculate the activities of the antioxidant enzymes.

### Determination of total thiols and non-protein thiol contents of *D. melanogaster* exposed to rotenone and TA

The levels of total and non-protein thiols were determined based on the method of Ellman^52^. The reaction mixture was made up of 170 μL of 0.1 M potassium phosphate buffer (pH 7.4), 20 μL of sample, and 10 μL of DTNB. The reaction was allowed to incubate for 30 min at room temperature, and the absorbance was measured at 412 nm in a SpectraMax microplate reader (Molecular devices). To determine the non-protein thiol content, samples were precipitated with 4% sulphosalicyclic acid (4%, ratio 1:1) and centrifuged for 10 min at 5000 rpm at 4 °C. The assay mixture for non-protein thiols consisted of 550 µL of 0.1 M phosphate buffer, 100 µL of supernatant and 100 µL of DTNB. For both total and non-protein thiols, GSH was used as standard, and the data were expressed as in μmol/mg of protein.

### Determination of glutathione-S-transferase activity of *D. melanogaster* exposed to rotenone and TA

The GST activity was assessed according to Habig and Jakoby^53^ with the use of 1-chloro-2,4-dinitrobenzene (CDNB) as substrate. The reaction mixture was contained 270 μL of solution A (20 mL of 0.25 M potassium phosphate buffer, pH 7.0, with 2.5 mM EDTA, 10.5 mL of distilled water and 500 μL of 0.1 M GSH at 25 °C), 20 μL of the sample (1:5 dilution), and 10 μL of 25 mM CDNB. The reaction was monitored at 340 nm for 5 min at 10 s intervals in a SpectraMax microplate reader (Molecular devices). The data were expressed as μmol/min/mg protein.

### Determination of catalase activity of *D. melanogaster* exposed to rotenone and TA

The activity of catalase was carried out using the method of Aebi^54^. This was based on the clearance of hydrogen peroxide in the presence of catalase. The reaction mixture was made up of 1800 μL of 50 mM phosphate buffer (pH 7.0), 180 μL of 300 mM H_2_O_2_, and 20 μL of sample (1:50 dilution). The loss in absorbance of hydrogen peroxide was monitored at 240 nm, for 2 min at 10 s intervals and at 25 °C with a UV/Visible spectrophotometer. The catalase activity was expressed as μmol of H_2_O_2_ consumed/min/mg protein.

### Determination of hydrogen peroxide level of *D. melanogaster* exposed to rotenone and TA

The protocol by Wolff^55^ was used to estimate H_2_O_2_ level. The reaction mixture contained 590 μL of FOX-1 (Ferrous Oxidation-Xylenol orange) reagent and 10 μL of sample. This was followed by 30 min incubation at room temperature, and the absorbance measured at 560 nm. The concentration of hydrogen peroxide generated was determined using extinction coefficient of H_2_O_2_ and expressed as μmol/mL.

### Determination of nitric oxide (nitrate/nitrite) level of *D. melanogaster* exposed to rotenone and TA

Nitric oxide (nitrate and nitrite) was quantified using Griess reaction following the protocol described at Green et al*.*^56^. The fly homogenates were incubated in Griess reagent at room temperature for 20 min, and the absorbance measured at 550 nm. The concentration of NO in the samples were calculated using the standard calibration curve of NaNO_2_ and expressed as μmol/L*.*

### Determination of acetylcholinesterase (AChE) activity of *D. melanogaster* exposed to rotenone and TA

AChE activity was determined with the method of Ellman et al*.*^57^ The reaction contained 135 μL of distilled water, 20 μL of 100 mM potassium phosphate buffer (pH 7.4), 10 mM DTNB (20 μL), 5 μL of sample and 20 μL of 8 mM acetylthiocholine as initiator. The reaction was monitored for 5 min at 15 s intervals at 412 nm using a SpectraMax microplate reader (Molecular devices). The enzyme activity was estimated as μmol of acetylthiocholine hydrolyzed/min/mg protein.

### Determination of malondialdehyde level of *D. melanogaster* exposed to rotenone and TA

Lipid peroxidation was determined as previously discussed by Ohkawa et al.^58^ by measuring the formation of Thiobarbituric Acid Ractive Substances (TBARS). The reaction mixture contained of 5 µL of 10 mM Butyl-hydroxytoluene (BHT), 200 µL of 0.67% thiobarbituric acid, 600 µL of 1% O-phosphoric acid, 105 µl of distilled water and 90 µL of supernatant. The mixture was incubated at 90 °C for 45 min and the absorbance was measured at 535 nm in a spectrophotometer. The data were expressed as μmol of MDA/L.

### Determination of protein carbonyl content of *D. melanogaster* exposed to rotenone and TA

Estimation of Protein Carbonyl (PC) content was done following the method of Dalle-Donne et al*.*^59^. The samples were added to trichloroacetic acid (20%) to precipitate the proteins. The reaction of the carbonyl group with 2, 4-dinitrophenylhydrazine to yield a stable dinitrophenylhydrazone. This was then mixed with guanidine hydrochloride (6 M) and the absorbance was measured at 375 nm. Protein Carbonyl content was quantified using the molar absorption coefficient of 22,000 M^−1^ cm^−1^.

### Determination of mitochondrial metabolic rate (cell viability) of *D. melanogaster* exposed to rotenone and TA

Mitochondrial metabolic rate (cell viability) was determined based on enzymatic reduction of MTT (3-(4,5-dimethylthiazol-2-yl)-2,5-diphenyltetrazolium bromide) to MTT-formazan in flies at a final concentration of 5 mg/mL according to Abe and Matsuki^58^. The data were expressed as a percentage of the control.

### Measurement of emergence rate of *D. melanogaster* exposed to rotenone and TA

The emergence rate of the progenies of flies into adulthood were also investigated after parent flies were treated with both TA and/or rotenone as previously reported^4,9^. Briefly, 1 to 3 days old of 10 males and 10 female flies/ vial (5 replicates) were placed in diets containing similar doses of rotenone and TA as described above for 24 h. The flies were then removed from the diets while the embryos were allowed to develop to adulthood. The number of newly emerged flies from each vial were then recorded over a period of 2 weeks and expressed as percentage of control.

### Molecular docking

Molecular docking analysis was performed to further investigate the anti-inflammatory capacity of TA by determining its binding affinity for some inflammatory protein targets (TNF-α, Caspase-1 and Cox-2) as compared to their respective standard inhibitors (Rofecoxib, Pralnacasan and Thalidomide). The grid box was set based on the protein structural dimensions and each ligand was set to have eight exhaustive conformations before being forwarded for docking using auto-dock vina in the PyRx workspace. The 3D structures of the protein–ligand complexes were prepared using the Chimera 1.14 workspace, and this was followed by visualization of the ligand–protein interactions and generation of the 2D structure using the Discovery Studio 2020.

### Ligands and protein mining

The structure data file (SDF) format for the natural compound (TA) and inhibitors of TNF-α (Rofecoxib), Caspase-1 (Pralnacasan) and Cox-2 (Thalidomide) were downloaded from the PubChem database. The 3D crystal structures, also in SDF format, for TNF-α (PDB ID: 2AZ5), Caspase-1 (PDB ID: 1ICE) and Cox-2 (PDB ID: 5KIR) of humans (*Homo sapiens*) were obtained from the RCSB protein data bank (PDB), whereas the amino acid sequence of the protein homologues of the same protein targets for *D. melanogaster* were obtained from the UniProt webserver and their homology models were generated using the SWISS-MODEL webserver.

### Ligand and protein preparation

Individual 3D structures of the selected protein targets were uploaded onto Chimera 1.14 workspace in order to prepare them for docking and they were converted into PDBQT format using the PyRx software. The ligands were also imported onto the PyRx workplace accordingly and converted to PDBQT format using the Open Babel plugin. Then, the proteins and ligands in PDBQT format were selected and forwarded for grid box generation.

### Statistical analysis

Data were expressed as mean ± standard deviation. One-way ANOVA (Analysis of Variance) followed by Tukey’s post hoc test was utilized to calculate the significant differences among groups under various treatments. Statistically significant difference was set at *p* < 0.05, using the GraphPad Prism5.0 software.

## Data Availability

The datasets generated during and/or analysed during the current study are available from the corresponding author on reasonable request.
